# Ethyl 2-benzyl-1-propyl-1*H*-indole-3-carboxyl­ate

**DOI:** 10.1107/S1600536809016493

**Published:** 2009-05-14

**Authors:** Peifan Li, Wenjie Wang, Caiyun Li, Xiaoxia Bian

**Affiliations:** a Department of Pharmaceuticals, Tianjin Medical College, Tianjin 300222, People’s Republic of China

## Abstract

In the title compound, C_21_H_23_NO_2_, the dihedral angle between the indole ring system and the benzyl ring is 75.92 (9)°. The crystal packing is controlled by C—H⋯O and C—H⋯π inter­actions.

## Related literature

For the synthesis of the title compound, see: Du *et al.* (2006 ▶). For its precursor, see: Jin *et al.* (2009 ▶).
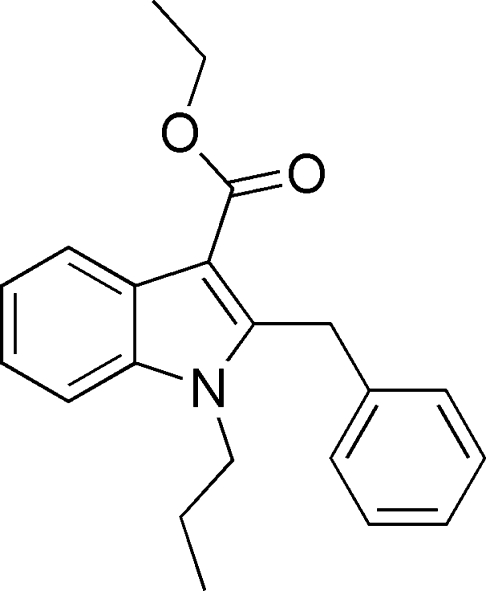


## Experimental

### 

#### Crystal data

C_21_H_23_NO_2_
*M* *_r_* = 321.40
Orthorhombic, 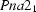

*a* = 16.231 (3) Å
*b* = 19.479 (4) Å
*c* = 5.5226 (11) Å
*V* = 1746.0 (6) Å^3^
*Z* = 4
Mo *K*α radiation
μ = 0.08 mm^−1^
*T* = 113 K
0.20 × 0.16 × 0.12 mm

#### Data collection

Rigaku Saturn CCD area-detector diffractometer
Absorption correction: multi-scan (*CrystalClear*; Rigaku/MSC, 2005 ▶) *T*_min_ = 0.985, *T*_max_ = 0.991
13765 measured reflections
2204 independent reflections
2060 reflections with *I* > 2σ(*I*)
*R*_int_ = 0.044

#### Refinement

*R*[*F* ^2^ > 2σ(*F* ^2^)] = 0.044
*wR*(*F* ^2^) = 0.102
*S* = 1.07
2204 reflections
220 parameters
1 restraint
H-atom parameters constrained
Δρ_max_ = 0.18 e Å^−3^
Δρ_min_ = −0.15 e Å^−3^

### 

Data collection: *CrystalClear* (Rigaku/MSC, 2005 ▶); cell refinement: *CrystalClear*; data reduction: *CrystalClear*; program(s) used to solve structure: *SHELXS97* (Sheldrick, 2008 ▶); program(s) used to refine structure: *SHELXL97* (Sheldrick, 2008 ▶); molecular graphics: *SHELXTL* (Sheldrick, 2008 ▶); software used to prepare material for publication: *CrystalStructure* (Rigaku/MSC, 2005 ▶).

## Supplementary Material

Crystal structure: contains datablocks global, I. DOI: 10.1107/S1600536809016493/hb2963sup1.cif

Structure factors: contains datablocks I. DOI: 10.1107/S1600536809016493/hb2963Isup2.hkl

Additional supplementary materials:  crystallographic information; 3D view; checkCIF report

## Figures and Tables

**Table 1:** Hydrogen-bond geometry (Å, °)

*D*—H⋯*A*	*D*—H	H⋯*A*	*D*⋯*A*	*D*—H⋯*A*
C2—H2⋯O1^i^	0.95	2.71	3.653 (3)	173
C9—H9*B*⋯O1^i^	0.99	2.88	3.680 (3)	138
C11—H11*A*⋯O1^i^	0.98	2.69	3.514 (3)	142
C12—H12*A*⋯O1	0.99	2.39	3.044 (3)	123
C18—H18⋯O1	0.95	2.96	3.620 (3)	128
C21—H21*A*⋯O2^ii^	0.98	2.91	3.555 (3)	124
C3—H3⋯*Cg*^iii^	0.95	2.82	3.632 (3)	144

Symmetry codes: (i) 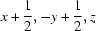; (ii) 
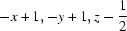
; (iii) 
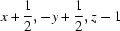
. *Cg* is the centroid of the C13–C18 ring.

**Fig. 1.**
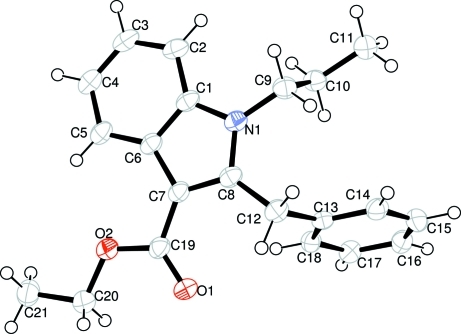
The molecular structure of molecule one of (I) with the atom-numbering scheme and 50% probability displacement ellipsoids.

## supplementary crystallographic information

### Comment

Indole chemistry continue to capture the attention of synthetic organic chemists due to indole's pharmaceutical properties. Recently we have reported the crystal structure of (*Z*)-ethyl 2,4-diphenyl-3-(propylamino)-but-2-enoate(Jin *et al.*, 2009). Starting from this precursor, its indole derivative was prepared according to the method of Du and coworkers. To further study the SAR, we determine the crystal structure of this indole derivative.

In the molecular structure,(I)(Fig. 1), the indole ring is coplanar with a dihedral angle of 0.21 (12)° between its pyrrole ring and fused benzene ring. The indole ring forms an angle of 75.92 (9)° with the benzyl ring.

### Experimental

The title compound was prepared according to the method of the literature (Du *et al.*, 2006). The crystals fit for X-ray diffraction were grown from a mixture of ethyl actate and petroleum ether.

### Refinement

All H atoms were positioned geometrically (C—H = 0.93–0.97 Å)and refined as riding with *U*_iso_(H) = 1.2*U*_eq_(CH and CH_2_) or 1.5*U*_eq_(CH_3_). Friedel Pairs were merged before refinement.

### Crystal data

**Table:**

C_21_H_23_NO_2_	*F*(000) = 688
*M**_r_* = 321.40	*D*_x_ = 1.223 Mg m^−^^3^
Orthorhombic, *P**n**a*2_1_	Mo *K*α radiation, λ = 0.71073 Å
Hall symbol: P 2c -2n	Cell parameters from 5126 reflections
*a* = 16.231 (3) Å	θ = 2.4–27.5°
*b* = 19.479 (4) Å	µ = 0.08 mm^−^^1^
*c* = 5.5226 (11) Å	*T* = 113 K
*V* = 1746.0 (6) Å^3^	Needle, colourless
*Z* = 4	0.20 × 0.16 × 0.12 mm

### Data collection

**Table:**

Rigaku Saturn CCD area-detector diffractometer	2204 independent reflections
Radiation source: rotating anode	2060 reflections with *I* > 2σ(*I*)
confocal	*R*_int_ = 0.044
Detector resolution: 7.31 pixels mm^-1^	θ_max_ = 27.5°, θ_min_ = 2.4°
ω and φ scans	*h* = −21→20
Absorption correction: multi-scan (*CrystalClear*; Rigaku/MSC, 2005)	*k* = −25→25
*T*_min_ = 0.985, *T*_max_ = 0.991	*l* = −4→7
13765 measured reflections	

### Refinement

**Table:**

Refinement on *F*^2^	Secondary atom site location: difference Fourier map
Least-squares matrix: full	Hydrogen site location: inferred from neighbouring sites
*R*[*F*^2^ > 2σ(*F*^2^)] = 0.044	H-atom parameters constrained
*wR*(*F*^2^) = 0.102	*w* = 1/[σ^2^(*F*_o_^2^) + (0.0496*P*)^2^ + 0.3147*P*] where *P* = (*F*_o_^2^ + 2*F*_c_^2^)/3
*S* = 1.07	(Δ/σ)_max_ < 0.001
2204 reflections	Δρ_max_ = 0.18 e Å^−^^3^
220 parameters	Δρ_min_ = −0.15 e Å^−^^3^
1 restraint	Extinction correction: *SHELXL*, Fc^*^=kFc[1+0.001xFc^2^λ^3^/sin(2θ)]^-1/4^
Primary atom site location: structure-invariant direct methods	Extinction coefficient: 0.044 (4)

### Special details

**Table:**

Geometry. All e.s.d.'s (except the e.s.d. in the dihedral angle between two l.s. planes) are estimated using the full covariance matrix. The cell e.s.d.'s are taken into account individually in the estimation of e.s.d.'s in distances, angles and torsion angles; correlations between e.s.d.'s in cell parameters are only used when they are defined by crystal symmetry. An approximate (isotropic) treatment of cell e.s.d.'s is used for estimating e.s.d.'s involving l.s. planes.
Refinement. Refinement of *F*^2^ against ALL reflections. The weighted *R*-factor *wR* and goodness of fit *S* are based on *F*^2^, conventional *R*-factors *R* are based on *F*, with *F* set to zero for negative *F*^2^. The threshold expression of *F*^2^ > σ(*F*^2^) is used only for calculating *R*-factors(gt) *etc*. and is not relevant to the choice of reflections for refinement. *R*-factors based on *F*^2^ are statistically about twice as large as those based on *F*, and *R*- factors based on ALL data will be even larger.

### Fractional atomic coordinates and isotropic or equivalent isotropic displacement parameters (Å^2^)

**Table:**

	*x*	*y*	*z*	*U*_iso_*/*U*_eq_	
O1	0.38761 (9)	0.34048 (8)	0.2851 (4)	0.0430 (5)	
O2	0.45086 (8)	0.40505 (7)	0.0042 (3)	0.0357 (4)	
N1	0.62712 (10)	0.24714 (8)	0.3130 (4)	0.0294 (4)	
C1	0.66004 (12)	0.28279 (10)	0.1183 (4)	0.0300 (5)	
C2	0.73763 (13)	0.27623 (11)	0.0133 (5)	0.0355 (5)	
H2	0.7768	0.2440	0.0718	0.043*	
C3	0.75495 (13)	0.31898 (11)	−0.1805 (5)	0.0392 (6)	
H3	0.8075	0.3164	−0.2557	0.047*	
C4	0.69695 (14)	0.36590 (11)	−0.2680 (5)	0.0383 (6)	
H4	0.7107	0.3941	−0.4023	0.046*	
C5	0.62019 (13)	0.37209 (10)	−0.1635 (5)	0.0339 (5)	
H5	0.5813	0.4042	−0.2245	0.041*	
C6	0.60052 (12)	0.33007 (10)	0.0348 (4)	0.0288 (4)	
C7	0.52909 (13)	0.32030 (9)	0.1883 (4)	0.0288 (5)	
C8	0.54835 (12)	0.26987 (10)	0.3565 (5)	0.0290 (4)	
C9	0.67057 (12)	0.19227 (10)	0.4394 (5)	0.0326 (5)	
H9A	0.6472	0.1873	0.6041	0.039*	
H9B	0.7293	0.2052	0.4568	0.039*	
C10	0.66510 (12)	0.12335 (10)	0.3092 (5)	0.0336 (5)	
H10A	0.6066	0.1103	0.2890	0.040*	
H10B	0.6901	0.1273	0.1464	0.040*	
C11	0.70976 (14)	0.06838 (11)	0.4534 (5)	0.0404 (6)	
H11A	0.7673	0.0821	0.4774	0.061*	
H11B	0.7079	0.0248	0.3647	0.061*	
H11C	0.6830	0.0627	0.6112	0.061*	
C12	0.49933 (13)	0.24338 (10)	0.5656 (4)	0.0315 (5)	
H12A	0.4530	0.2753	0.5963	0.038*	
H12B	0.5348	0.2433	0.7115	0.038*	
C13	0.46466 (12)	0.17145 (10)	0.5295 (4)	0.0271 (4)	
C14	0.47834 (13)	0.12068 (10)	0.7011 (4)	0.0317 (5)	
H14	0.5121	0.1302	0.8376	0.038*	
C15	0.44300 (14)	0.05585 (11)	0.6750 (5)	0.0360 (5)	
H15	0.4527	0.0214	0.7933	0.043*	
C16	0.39379 (13)	0.04180 (11)	0.4763 (5)	0.0350 (5)	
H16	0.3692	−0.0022	0.4589	0.042*	
C17	0.38041 (13)	0.09212 (10)	0.3027 (5)	0.0331 (5)	
H17	0.3470	0.0825	0.1656	0.040*	
C18	0.41579 (12)	0.15656 (10)	0.3295 (4)	0.0296 (4)	
H18	0.4065	0.1908	0.2100	0.036*	
C19	0.44951 (13)	0.35436 (10)	0.1705 (4)	0.0308 (5)	
C20	0.37460 (14)	0.44182 (11)	−0.0340 (6)	0.0410 (6)	
H20A	0.3565	0.4641	0.1180	0.049*	
H20B	0.3308	0.4099	−0.0879	0.049*	
C21	0.39091 (15)	0.49475 (12)	−0.2251 (5)	0.0442 (6)	
H21A	0.4351	0.5254	−0.1710	0.066*	
H21B	0.3407	0.5215	−0.2539	0.066*	
H21C	0.4076	0.4719	−0.3755	0.066*	

### Atomic displacement parameters (Å^2^)

**Table:**

	*U*^11^	*U*^22^	*U*^33^	*U*^12^	*U*^13^	*U*^23^
O1	0.0300 (8)	0.0423 (8)	0.0567 (13)	0.0019 (6)	0.0131 (9)	0.0115 (9)
O2	0.0274 (7)	0.0368 (8)	0.0430 (10)	−0.0020 (6)	0.0009 (8)	0.0075 (8)
N1	0.0260 (8)	0.0311 (8)	0.0312 (10)	−0.0032 (6)	0.0012 (8)	−0.0027 (8)
C1	0.0266 (10)	0.0306 (9)	0.0327 (12)	−0.0088 (8)	0.0015 (9)	−0.0073 (9)
C2	0.0254 (10)	0.0386 (10)	0.0426 (14)	−0.0055 (8)	0.0034 (10)	−0.0085 (10)
C3	0.0303 (11)	0.0443 (11)	0.0430 (15)	−0.0113 (9)	0.0102 (11)	−0.0075 (12)
C4	0.0378 (12)	0.0397 (11)	0.0375 (14)	−0.0123 (9)	0.0062 (11)	−0.0025 (11)
C5	0.0336 (11)	0.0316 (9)	0.0365 (12)	−0.0097 (8)	0.0031 (10)	−0.0032 (10)
C6	0.0280 (10)	0.0285 (9)	0.0299 (11)	−0.0067 (7)	0.0022 (9)	−0.0053 (9)
C7	0.0274 (10)	0.0270 (9)	0.0319 (12)	−0.0055 (7)	0.0028 (9)	−0.0027 (9)
C8	0.0268 (10)	0.0302 (9)	0.0299 (11)	−0.0059 (8)	0.0008 (9)	−0.0049 (9)
C9	0.0292 (10)	0.0361 (10)	0.0325 (12)	−0.0017 (8)	−0.0030 (10)	−0.0023 (10)
C10	0.0289 (10)	0.0353 (10)	0.0366 (13)	−0.0010 (8)	−0.0005 (10)	−0.0015 (10)
C11	0.0380 (12)	0.0375 (11)	0.0457 (14)	−0.0001 (9)	−0.0005 (12)	0.0050 (11)
C12	0.0324 (10)	0.0342 (10)	0.0278 (12)	−0.0020 (8)	0.0032 (9)	−0.0037 (9)
C13	0.0231 (9)	0.0308 (9)	0.0273 (11)	0.0012 (7)	0.0046 (8)	−0.0017 (9)
C14	0.0288 (10)	0.0400 (10)	0.0262 (11)	−0.0006 (9)	0.0007 (9)	0.0025 (10)
C15	0.0359 (11)	0.0382 (11)	0.0338 (12)	0.0039 (9)	0.0005 (10)	0.0059 (10)
C16	0.0388 (11)	0.0308 (9)	0.0352 (13)	−0.0024 (8)	0.0057 (10)	−0.0003 (10)
C17	0.0336 (11)	0.0373 (10)	0.0283 (12)	−0.0038 (8)	−0.0018 (10)	−0.0016 (10)
C18	0.0306 (10)	0.0313 (9)	0.0271 (11)	−0.0001 (8)	0.0008 (9)	0.0017 (9)
C19	0.0292 (10)	0.0280 (9)	0.0353 (12)	−0.0052 (8)	0.0025 (10)	−0.0018 (9)
C20	0.0327 (11)	0.0392 (11)	0.0512 (16)	0.0003 (9)	0.0010 (11)	0.0093 (12)
C21	0.0441 (13)	0.0411 (11)	0.0474 (16)	−0.0055 (10)	−0.0029 (12)	0.0099 (12)

### Geometric parameters (Å, °)

**Table:**

O1—C19	1.218 (3)	C10—H10B	0.9900
O2—C19	1.349 (3)	C11—H11A	0.9800
O2—C20	1.446 (3)	C11—H11B	0.9800
N1—C8	1.374 (3)	C11—H11C	0.9800
N1—C1	1.387 (3)	C12—C13	1.523 (3)
N1—C9	1.458 (3)	C12—H12A	0.9900
C1—C2	1.392 (3)	C12—H12B	0.9900
C1—C6	1.412 (3)	C13—C14	1.387 (3)
C2—C3	1.385 (4)	C13—C18	1.391 (3)
C2—H2	0.9500	C14—C15	1.394 (3)
C3—C4	1.398 (3)	C14—H14	0.9500
C3—H3	0.9500	C15—C16	1.384 (3)
C4—C5	1.378 (3)	C15—H15	0.9500
C4—H4	0.9500	C16—C17	1.388 (3)
C5—C6	1.404 (3)	C16—H16	0.9500
C5—H5	0.9500	C17—C18	1.388 (3)
C6—C7	1.449 (3)	C17—H17	0.9500
C7—C8	1.388 (3)	C18—H18	0.9500
C7—C19	1.455 (3)	C20—C21	1.499 (4)
C8—C12	1.494 (3)	C20—H20A	0.9900
C9—C10	1.526 (3)	C20—H20B	0.9900
C9—H9A	0.9900	C21—H21A	0.9800
C9—H9B	0.9900	C21—H21B	0.9800
C10—C11	1.518 (3)	C21—H21C	0.9800
C10—H10A	0.9900		
			
C19—O2—C20	116.62 (17)	C10—C11—H11C	109.5
C8—N1—C1	109.44 (18)	H11A—C11—H11C	109.5
C8—N1—C9	127.0 (2)	H11B—C11—H11C	109.5
C1—N1—C9	123.49 (17)	C8—C12—C13	114.42 (18)
N1—C1—C2	128.7 (2)	C8—C12—H12A	108.7
N1—C1—C6	108.43 (17)	C13—C12—H12A	108.7
C2—C1—C6	122.9 (2)	C8—C12—H12B	108.7
C3—C2—C1	116.8 (2)	C13—C12—H12B	108.7
C3—C2—H2	121.6	H12A—C12—H12B	107.6
C1—C2—H2	121.6	C14—C13—C18	119.03 (18)
C2—C3—C4	121.6 (2)	C14—C13—C12	120.5 (2)
C2—C3—H3	119.2	C18—C13—C12	120.43 (19)
C4—C3—H3	119.2	C13—C14—C15	120.6 (2)
C5—C4—C3	121.4 (2)	C13—C14—H14	119.7
C5—C4—H4	119.3	C15—C14—H14	119.7
C3—C4—H4	119.3	C16—C15—C14	119.9 (2)
C4—C5—C6	118.8 (2)	C16—C15—H15	120.1
C4—C5—H5	120.6	C14—C15—H15	120.1
C6—C5—H5	120.6	C15—C16—C17	119.9 (2)
C5—C6—C1	118.65 (19)	C15—C16—H16	120.1
C5—C6—C7	135.6 (2)	C17—C16—H16	120.1
C1—C6—C7	105.70 (19)	C18—C17—C16	120.0 (2)
C8—C7—C6	107.73 (18)	C18—C17—H17	120.0
C8—C7—C19	124.58 (19)	C16—C17—H17	120.0
C6—C7—C19	127.7 (2)	C17—C18—C13	120.6 (2)
N1—C8—C7	108.7 (2)	C17—C18—H18	119.7
N1—C8—C12	121.3 (2)	C13—C18—H18	119.7
C7—C8—C12	129.92 (19)	O1—C19—O2	121.99 (19)
N1—C9—C10	113.02 (19)	O1—C19—C7	126.6 (2)
N1—C9—H9A	109.0	O2—C19—C7	111.41 (18)
C10—C9—H9A	109.0	O2—C20—C21	106.99 (19)
N1—C9—H9B	109.0	O2—C20—H20A	110.3
C10—C9—H9B	109.0	C21—C20—H20A	110.3
H9A—C9—H9B	107.8	O2—C20—H20B	110.3
C11—C10—C9	110.2 (2)	C21—C20—H20B	110.3
C11—C10—H10A	109.6	H20A—C20—H20B	108.6
C9—C10—H10A	109.6	C20—C21—H21A	109.5
C11—C10—H10B	109.6	C20—C21—H21B	109.5
C9—C10—H10B	109.6	H21A—C21—H21B	109.5
H10A—C10—H10B	108.1	C20—C21—H21C	109.5
C10—C11—H11A	109.5	H21A—C21—H21C	109.5
C10—C11—H11B	109.5	H21B—C21—H21C	109.5
H11A—C11—H11B	109.5		
			
C8—N1—C1—C2	−180.0 (2)	C6—C7—C8—C12	175.6 (2)
C9—N1—C1—C2	−2.4 (3)	C19—C7—C8—C12	−6.3 (4)
C8—N1—C1—C6	0.2 (2)	C8—N1—C9—C10	96.0 (3)
C9—N1—C1—C6	177.74 (19)	C1—N1—C9—C10	−81.1 (2)
N1—C1—C2—C3	−179.6 (2)	N1—C9—C10—C11	−178.64 (18)
C6—C1—C2—C3	0.2 (3)	N1—C8—C12—C13	−75.8 (3)
C1—C2—C3—C4	−0.7 (3)	C7—C8—C12—C13	107.9 (2)
C2—C3—C4—C5	0.6 (4)	C8—C12—C13—C14	127.6 (2)
C3—C4—C5—C6	−0.1 (3)	C8—C12—C13—C18	−55.3 (3)
C4—C5—C6—C1	−0.4 (3)	C18—C13—C14—C15	−0.6 (3)
C4—C5—C6—C7	−179.1 (2)	C12—C13—C14—C15	176.5 (2)
N1—C1—C6—C5	−179.83 (18)	C13—C14—C15—C16	0.0 (3)
C2—C1—C6—C5	0.3 (3)	C14—C15—C16—C17	0.6 (3)
N1—C1—C6—C7	−0.8 (2)	C15—C16—C17—C18	−0.5 (3)
C2—C1—C6—C7	179.4 (2)	C16—C17—C18—C13	−0.1 (3)
C5—C6—C7—C8	179.9 (2)	C14—C13—C18—C17	0.7 (3)
C1—C6—C7—C8	1.1 (2)	C12—C13—C18—C17	−176.49 (19)
C5—C6—C7—C19	1.8 (4)	C20—O2—C19—O1	−0.7 (3)
C1—C6—C7—C19	−177.0 (2)	C20—O2—C19—C7	178.6 (2)
C1—N1—C8—C7	0.5 (2)	C8—C7—C19—O1	−5.9 (4)
C9—N1—C8—C7	−176.92 (19)	C6—C7—C19—O1	171.8 (2)
C1—N1—C8—C12	−176.43 (18)	C8—C7—C19—O2	174.8 (2)
C9—N1—C8—C12	6.1 (3)	C6—C7—C19—O2	−7.5 (3)
C6—C7—C8—N1	−1.0 (2)	C19—O2—C20—C21	−179.8 (2)
C19—C7—C8—N1	177.15 (19)		

### Hydrogen-bond geometry (Å, °)

**Table:**

*D*—H···*A*	*D*—H	H···*A*	*D*···*A*	*D*—H···*A*
C2—H2···O1^i^	0.95	2.71	3.653 (3)	173
C9—H9B···O1^i^	0.99	2.88	3.680 (3)	138
C11—H11A···O1^i^	0.98	2.69	3.514 (3)	142
C12—H12A···O1	0.99	2.39	3.044 (3)	123
C18—H18···O1	0.95	2.96	3.620 (3)	128
C21—H21A···O2^ii^	0.98	2.91	3.555 (3)	124
C3—H3···Cg^iii^	0.95	2.82	3.632 (3)	144

Symmetry codes: (i) *x*+1/2, −*y*+1/2, *z*; (ii) −*x*+1, −*y*+1, *z*−1/2; (iii) *x*+1/2, −*y*+1/2, *z*−1.
